# Variability of ENSO Forecast Skill in 2‐Year Global Reforecasts Over the 20th Century

**DOI:** 10.1029/2022GL097885

**Published:** 2022-05-18

**Authors:** Antje Weisheimer, Magdalena A. Balmaseda, Tim N. Stockdale, Michael Mayer, S. Sharmila, Harry Hendon, Oscar Alves

**Affiliations:** ^1^ European Centre for Medium‐Range Weather Forecasts (ECMWF) Reading UK; ^2^ University of Oxford Department of Physics National Centre for Atmospheric Science (NCAS) Oxford UK; ^3^ Department of Meteorology and Geophysics University of Vienna Vienna Austria; ^4^ Centre for Applied Climate Sciences University of Southern Queensland Toowoomba Australia; ^5^ Bureau of Meteorology Melbourne Australia; ^6^ Monash University Melbourne Australia

**Keywords:** ENSO predictability, seasonal forecasting, climate variability

## Abstract

In order to explore temporal changes of predictability of El Niño Southern Oscillation (ENSO), a novel set of global biennial climate reforecasts for the historical period 1901–2010 has been generated using a modern initialized coupled forecasting system. We find distinct periods of enhanced long‐range skill at the beginning and at the end of the twentieth century, and an extended multi‐decadal epoch of reduced skill during the 1930s–1950s. Once the forecast skill extends beyond the first spring barrier, the predictability limit is much enhanced and our results provide support for the feasibility of skillful ENSO forecasts up to 18 months. Changes in the mean state, variability (amplitude), persistence, seasonal cycle and predictability suggest that multi‐decadal variations in the dynamical characteristics of ENSO rather than the data coverage and quality of the observations have primarily driven the reported non‐monotonic skill modulations.

## Key Points

- Novel 2‐year‐long global coupled retrospective forecast ensemble from 1901 to 2010 to study predictability of El Niño Southern Oscillation
- Periods of very high skill at the beginning and end of the twentieth century but extended multi‐decadal mid‐century epoch of reduced skill
- Non‐monotonic skill modulations cannot be explained by better recent observations and are likely linked to background changes instead

## 1 Introduction

El Niño Southern Oscillation (ENSO), the large‐scale fluctuation of the equatorial atmosphere and ocean over the tropical Pacific region with widespread climatic consequences, is arguably the most predictable climate mode at seasonal timescales and provides the scientific basis for global seasonal climate predictions (Barnston et al., 2010; Shukla et al., 2000; Timmermann et al., 2018; Trenberth et al., 1998). With significant progress made during the last decades in our understanding of the complexity of ENSO, the development of the observing systems, coupled general circulation models and data assimilation techniques for improved forecast initialization schemes, current forecast models can provide effective predictions of ENSO warm and cold events 6–12 months ahead (Barnston et al., 2019; Jin et al., 2008; Johnson et al., 2019; Tang et al., 2018; Tippett et al., 2019; Weisheimer et al., 2009).

However, the challenge of forecasting ENSO is not solved yet. Estimates of ENSO predictability are commonly based on retrospective forecasts (reforecasts) for the last two or three decades. This results in an overall small sample size of ENSO events with substantial uncertainties in skill estimates. In addition, decadal‐scale changes in the Pacific background state can make ENSO development in certain decades less predictable than others (Kirtman & Schopf, 1998). These background fluctuations can be seen as spontaneously generated multi‐decadal variations in ENSO diversity (Wang et al., 2019; Wittenberg et al., 2014). For example, changes of the tropical Pacific mean state related to the equatorial thermocline have been suggested as a source of interdecadal modulations of the amplitude of interannual variability (Gu & Philander, 1997) with an intensification of the ENSO signal observed in the second half of the twentieth Century.

How can changes in the ENSO amplitude affect its predictability? Kirtman and Schopf (1998) proposed a relationship between the amplitude of interannual variability, forecast skill, and limit of predictability in idealized experiments with a simple coupled model of the tropical Pacific and atmosphere: during decades with a stronger ENSO signal, the forecast skill is higher and the predictability limit longer than during periods with lower amplitudes of interannual variability. This behavior can be explained with the classical theory of the delayed oscillator mechanism which robustly maintains self‐sustained oscillations that drive sea surface temperature (SST) anomalies during high‐predictability periods. Due to the delayed oscillator's chaotic yet deterministic nature, predictability is largely limited by the growth of initial errors, and the associated potential forecast lead time is likely to be of the order of years. On the other hand, stochastic noise from the atmosphere, for example, westerly wind bursts, can influence the SST anomalies more strongly during epochs when the delayed oscillator is damped by cold SST and easterly wind stress anomalies, leading to reduced predictability.

A first attempt to reforecast historical ENSO events was made by Chen et al. (2004) who run the ENSO model of intermediate complexity of Zebiak and Cane (1987) from 1857 to 2003 by initializing the simulations with reconstructed SST data. They showed that ENSO predictability depends on the time period over which it is estimated: periods with high skill were dominated by strong ENSO events. Large El Niño events were found to be predictable up to 2 years in advance, raising the prospect of skillful long‐lead forecasts of ENSO. These results are consistent with the notion that the predictability of ENSO may reside in the prediction of its mean amplitude and not the uncertainty or spread (Kumar & Hu, 2014).

In this paper we move these early studies with simplified models and initialization techniques forward to explore ENSO predictability during the twentieth century in a state‐of‐the‐art dynamical seasonal forecasting system based on an initialized fully coupled global circulation model. CERA‐20C (Laloyaux et al., 2018), the Coupled European Reanalysis of the twentieth century from 1901 to 2010, provides an ideal opportunity to initialize long‐range reforecast experiments and to study state‐dependent predictability over an unprecedented 110‐year period. We introduce a new biennial (24‐month long) reforecast experiment with a lower‐resolution version of the European Centre for Medium‐Range Weather Forecasts (ECMWF) operational seasonal prediction system SEAS5 (Johnson et al., 2019) for the CERA‐20C period and label these reforecasts as SEAS5‐20C, see also Weisheimer et al. (2021). The purpose of SEAS5‐20C is twofold: (a) to examine the predictability of ENSO in the presence of multi‐decadal climate change and variability in long continuous reforecasts, and (b) to assess the feasibility of extending skillful seasonal forecasts to longer lead times by exploring the predictability limit of ENSO up to 2 years. As such SEAS5‐20C substantially extends and advances previous work of reforecasts run over a shorter historical period (Huang, Shin, et al., 2017) or with shorter forecast lead times (Weisheimer et al., 2020). New insight obtained through these experiments is expected to be valuable for the development and interpretation of future ENSO prediction systems and their associated uncertainties.

The paper is structured as follows: Section 2 describes the reforecast experiments, data and metrics. In Section 3 we analyze ENSO forecast skill and its variations throughout the hindcast period. Section 4 contrasts the ENSO mean state and variability in the model reforecasts with those from the verification, and in Section 5 we discuss and conclude our results.

## 2 Model Experiments, Data, and Skill Metrics

The CERA‐20C reanalysis aimed at reconstructing the weather and climate of the coupled atmosphere, ocean, land, ocean waves and sea‐ice system for the past period 1901–2010 (Laloyaux et al., 2018). It is used here to provide initial conditions (ICs) for coupled reforecast experiments, and its SST and atmospheric analysis provides the verification data for our ENSO predictions. CERA‐20C assimilated observed subsurface temperature and salinity profiles in the ocean and only conventional surface observations (surface pressure and marine winds) in the atmosphere. No satellite‐derived data went into the reanalysis. The SSTs were relaxed toward monthly HadISST2 observed data (Titchner & Rayner, 2014). In addition, verification of the ENSO predictions was also performed against ERSSTv5 data (Huang, Thorne, et al., 2017), see Supporting Information S1.

The reforecast experiments were performed with a low‐resolution configuration of ECMWF's operational fully coupled forecasting system SEAS5 (Johnson et al., 2019), labeled SEAS5‐20C. The experiments were run at atmospheric resolution T_co_199 (approximately 50 km) horizontally with 91 vertical levels and with a 1‐degree horizontal ocean resolution (with equatorial meridional refinement) using 42 vertical levels. Historical reconstructions of greenhouse gases (CMIP5) and volcanic stratospheric sulfate aerosol (GISS) were used as radiative forcings similar to SEAS5. The reforecasts have an ensemble size of 10 members, sampling the 10 CERA‐20C realizations of ocean ICs. SEAS5‐20C reforecasts were started on each 1 November and 1 May from 1901 to 2010 and have a forecast length of 24 months.

Throughout the paper we use the NINO3.4 SST index (Trenberth, 1997) defined as the average SST over the central equatorial Pacific (5°N–5°S, 170°–120°W) and the atmospheric equatorial Southern Oscillation Index (SOI) defined as the difference in standardized mean sea‐level pressure between the equatorial western Pacific area (5°N–5°S, 90°–140°E) and an equatorial eastern Pacific area (5°N–5°S, 130°–80°W) as a proxy of ENSO.

As a metric of the skill of the forecasts the ensemble‐mean anomaly correlation coefficient (*ACC*) will be used. It estimates the interannual correlation over *N* forecast years of the ensemble mean forecast anomaly and the corresponding observed anomaly. For ensemble‐mean forecast $\bar{FC_{i}}$ and observation $\mathrm{OB}S_{i}$ pairs with *i = 1 … N*, the *ACC* is defined as

(1) $$\mathrm{ACC}=\;\frac{\frac{1}{N}\sum_{i=1}^{N}{\bar{\mathrm{FC}}}_{i}^{\prime }*\mathrm{OB}S_{i}^{\prime }}{\sqrt{\frac{1}{N}\sum_{i=1}^{N}{{\bar{\mathrm{FC}}}_{i}^{\prime }}^{2}*\frac{1}{N}\sum_{i=1}^{N}\mathrm{OB}{S_{i}^{\prime }}^{2}}}$$

with ${\bar{\mathrm{FC}}}_{i}^{\prime }={\bar{\mathrm{FC}}}_{i}-\frac{1}{N}\overset{N}{\underset{i=1}{\sum }}{\bar{\mathrm{FC}}}_{i}$ and $\mathrm{OB}S_{i}^{\prime }=\mathrm{OB}S_{i}-\frac{1}{N}\overset{N}{\underset{i=1}{\sum }}\mathrm{OB}S_{i}$ being the ensemble‐mean forecast and observed anomalies, respectively. The ensemble‐mean forecast $\bar{FC_{i}}\;$ is given by ${\bar{\mathrm{FC}}}_{i}=\frac{1}{M}\overset{M}{\underset{j=1}{\sum }}FC_{i,j}$ where $FC_{i,j}$ with *j = 1… M* indicates the individual ensemble members of the forecast at year *i*.

The “*perfect model*” is defined as the concept where instead of observations, each individual ensemble member is, in turn, used as the verification so that the “*perfect model*” tries to forecast itself. The skill of the “*perfect model*” which is not affected by model biases, is also called “*potential skill*” and can be interpreted as the model's estimate of realizable skill in the hypothetical world where the statistics of the observations are identical to the statistics of the model predictions (Kumar et al., 2014). In analogy to Equation 1, the perfect model anomaly correlation coefficient *ACC*_*PM*_ is thus defined as

(2) $$\mathrm{AC}C_{\mathrm{PM}}=\;\frac{1}{M}\sum_{j=1}^{M}\frac{\frac{1}{N}\sum_{i=1}^{N}{\bar{\mathrm{FC}}}_{i}^{\prime }*FC_{i,j}^{\prime }}{\sqrt{\frac{1}{N}\sum_{i=1}^{N}{{\bar{\mathrm{FC}}}_{i}^{\prime }}^{2}*\frac{1}{N}\sum_{i=1}^{N}F{C_{i,j}^{\prime }}^{2}}}$$

## 3 ENSO Forecast Skill

**Figure 1:**
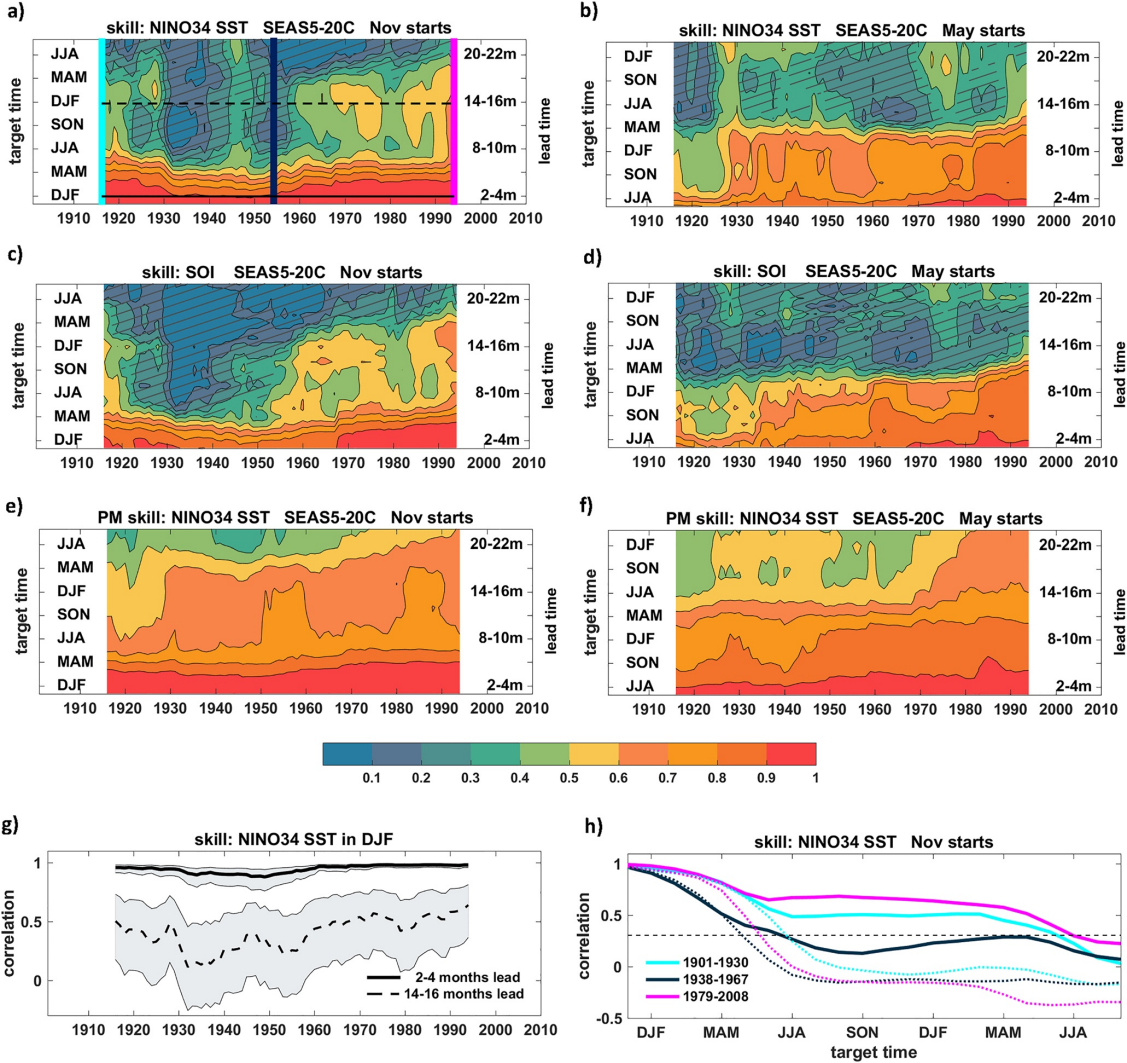
ENSO ensemble‐mean correlation skill of SEAS5‐20C with CERA‐20C as a function of hindcast period on the horizontal axis and forecast lead time on the vertical axis for 1 November (left) and 1 May (right) forecast start dates. (a) and (b) NINO3.4 SST. The solid and dashed horizontal lines indicate cross‐sectioned time series of skill shown in (g). The cyan, dark blue and magenta vertical lines indicate cross‐sectioned skill as a function of forecast lead time shown in (h). (c) and (d) Southern Oscillation Index (SOI). (e) and (f) NINO3.4 SST perfect model skill. (g) NINO3.4 SST correlation skill in DJF as a function of hindcast period for two distinct lead times. (h) NINO3.4 SST correlation skill as a function of target time for three distinct periods in SEAS5‐20C (solid lines) and for simple persistence forecasts (dotted lines). The dashed black line indicates the significance level at *α* = 0.05. For (a)–(g), skill is estimated for 30‐year moving windows and plotted at the central year. Hatching in (a)–(f) indicates non‐significant skill at *α* = 0.05.

The historical evolution of reforecast skill (ACC, see Equation 1) in predicting seasonal‐mean ENSO anomalies as a function of the hindcast period and forecast lead time is shown in Figure 1 for start dates in November (left) and May (right). Here, the skill has been computed for 30‐year windows moved across the period 1901–2010 by 1 year, with the resulting scores plotted at the central year of the corresponding window. Significance of the skill is estimated using a one‐sided *t*‐test at significance level *α* = 0.05. Figures 1a and 1b display the skill of NINO3.4 SSTs, whereas Figures 1c and 1d show the corresponding skill of the atmospheric manifestation of ENSO through the SOI.

Distinct periods of enhanced and statistically significant skill stand out against extended multi‐decadal epochs of reduced skill. On the shortest seasonal forecast lead time scales, skill during DJF in Figure 1a (see also Figure 1g for a cross‐section at forecast lead times 2–4 months) remains both robust and steadily high from the 1960s onwards with correlations above 0.95, in agreement with recent findings (Huang, Shin, et al., 2017; Liu et al., 2021; Weisheimer et al., 2020). The period between the 1930s and 1950s saw a decline in seasonal forecast skill and an increase in uncertainty. Interestingly, skill recovered in the very early decades of the century to reach similarly high values as during the most recent period, suggesting that the reduced skill in the mid‐twentieth century period is not simply due to poorer quality of the ICs compared to the later periods.

Significant skill up to lead times of approximately 18 months is found for November initialization from the late 1960s onward and, although to a slightly reduced degree, during the first two decades of the twentieth century. However, during the extended period from the 1920s to the 1960s the skill on all lead times is markedly reduced: once the forecasts hit the first boreal spring season and its associated predictability barrier (Liu et al., 2019; Webster & Yang, 1992), skill drops sharply and becomes non‐significant. The time series of NINO3.4 SSTs during DJF for forecast lead times 14–16 months is shown in Figure 1g and demonstrates the overall skill levels and their variations for forecasts started the previous November. We note that the decadal skill modulations are not sensitive to the choice of the observational data set, and similar results are obtained by verifying against ERSSTv5, see Figure S1 in Supporting Information S1.

Multi‐decadal variations in ENSO forecast skill are not exclusively related to SST predictions and can also be found in the ACC characteristics for the large‐scale atmospheric sea‐level pressure based Southern Oscillation Index (SOI, Figures 1c and 1d), supporting the conclusion that the ENSO skill variations are robust in both the surface temperatures of the equatorial Pacific and the atmosphere above.

The “*perfect model*” approach provides a testbed for analyzing how well the model can predict itself, and, because of its independence from observations, is sometimes interpreted as potential skill (Kumar et al., 2014). Figures 1e and 1f showing the perfect model *ACC*_*PM*_ (see Equation 2) for both start dates illustrate several interesting points: (a) The skill levels are, in general, higher than for the real‐world cases in Figures 1a and 1b indicating that the real‐world prediction skill is hampered by initialization and model formulation problems which indicate some potential for improvements; and (b) the multi‐decadal variability of perfect model skill is much smoother and temporally not coherent with the *ACC* real‐world skill variations. The skill reduction seen between 1930 and 1950 is not present in the perfect model skill, implying that it is an issue related to performance compared to observations and not to the intrinsic predictability in the model itself. The reasons for the discrepancy in decadal variations between the perfect model and observed skill can reside on deficiencies in both model and observations alike. We return to this point later in Section 4 when discussing possible drivers of decadal variations of skill.

The variations of forecast skill throughout the century are further illustrated in Figure 1h showing the lead‐time dependent skill evolution for three distinct 30‐year reforecast periods together with a benchmark persistence forecast started in November. Here, persistence means the SST anomaly of the ICs has been persisted throughout the forecast. The dynamical forecasts have higher skill during early and late century periods, but noticeably reduced skill from the start of the forecasts during the period 1931–1960. For all three periods skill remains roughly constant for approximately 12 months after the first spring, at levels between 0.4 and 0.7, extending skillful predictions to at least 18 months for certain periods (see also Dunstone et al., 2016). The skill of persistence forecasts for all three epochs falls to zero already after the first spring season, with persistence during the middle period showing a similar rapid skill loss during the first forecast months as the dynamical model.

The reforecasts started in May as shown in Figures 1b, 1d, and 1f also experience substantial multi‐decadal modulations of skill, although their temporal variations and lead‐time dependence differ from the November reforecasts. The predictability horizon of approx. twelve months is primarily determined by the spring predictability barrier. It is again the most recent period that has the highest skill over the longest lead time, but skill is quickly lost before the 1930s. Forecasts from May are, in general, more difficult because smaller SST anomalies provide weaker constraints on the ICs than for the winter peak ENSO season.

Using moving windows of 20 instead of 30 years for the skill estimation results in overall very consistent patterns, see Figures S2 and S3 in Supporting Information S1. Shorter windows can better detect abrupt skill changes but also introduce more noise into the correlation estimates.

**Figure 2:**
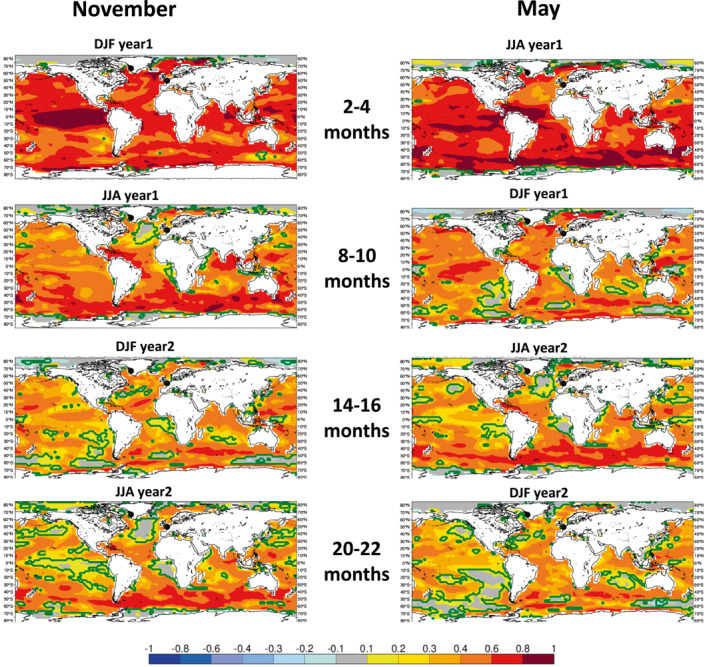
SST correlation skill of SEAS5‐20C with CERA‐20C over the hindcast period 1901–2010 for different forecast lead times initialized on 1 November (left) and 1 May (right). Green contours indicate where the skill becomes significant at *α* = 0.05.

In order to demonstrate the global SST forecast skill of the SEAS5‐20C reforecasts, Figure 2 shows the ACC over the full 110‐year hindcast period from 1901 to 2010 for different forecast lead times and both start dates. The skill patterns during the first year of the forecast resemble well the skill of ECMWF's operational system SEAS5 (Johnson et al., 2019). While the ENSO skill is substantially reduced on longer lead times beyond 1 year, some areas in the tropical Atlantic and Indian Oceans as well as in the Southern Ocean are skillfully predicted even up to 2 years. We also note that the skill patterns are common for forecasts verifying on a given season, irrespective of the lead time (see for instance forecast verifying in DJF, when ENSO peaks). This suggests that the skill is largely determined by the seasonal phase locking of ENSO (Jin et al., 1996), which is in turn influenced by the properties of the background state.

## 4 Variations of Mean State and ENSO Amplitude

What could be potential reasons for the pronounced multi‐decadal variability of forecast skill throughout the twentieth century? A simple hypothesis could be that the improved data coverage and quality of the observations in more recent decades compared to poorer coverage and perhaps quality at the beginning of the twentieth century, would lead to a reduction in forecast skill the further back in time we go. However, this is clearly not the case, as shown in Figure 1. Instead, the skill variations are highly non‐monotonic and the skill levels reached during the early decades of the century are similar in magnitude as for the recent well‐observed period. In addition, the skill variability of simple persistence forecasts based on observed anomalies, a measure that is independent of our model and gives highest persistence skill for the early century decades (see Figure 1h), provides further evidence against the data coverage and quality conjecture.

An alternative hypothesis is the following: The background state of the coupled ocean‐atmosphere system that defines ENSO underwent changes that led to the varying skill levels. Since the ACC skill is based on forecast and observed anomalies with respect to the mean state, any such background changes would need to have affected the forecasts in a non‐linear way, and would thus not be fully removed by using anomalies.

**Figure 3:**
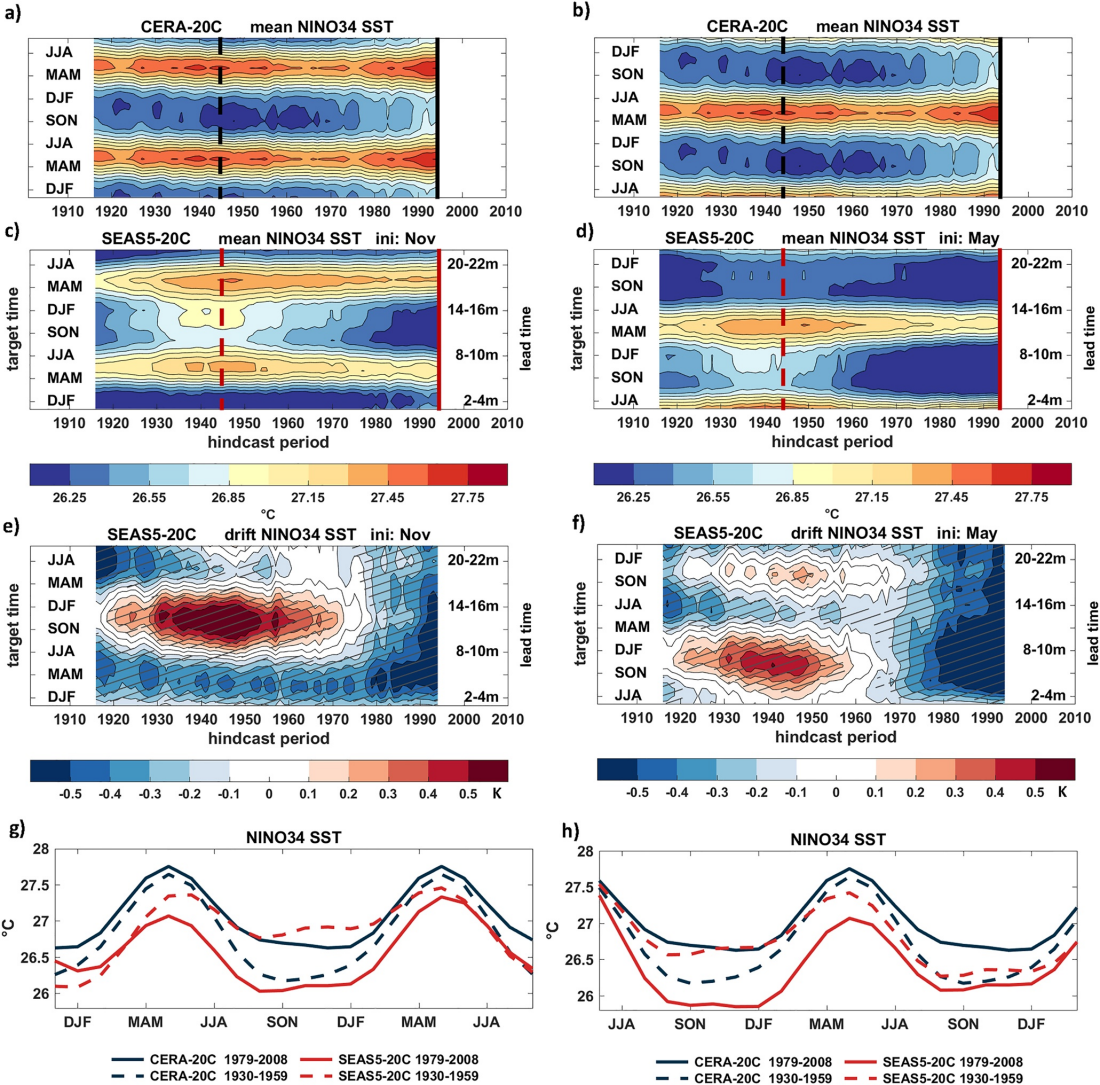
NINO3.4 SST mean‐state variations in CERA‐20C and SEAS5‐20C as a function of hindcast period and season/forecast lead time for 1 November (left) and 1 May (right) forecast start dates. (a) and (b): CERA‐20C. (c) and (d): SEAS5‐20C. (e) and (f) Bias SEAS5‐20C minus CERA‐20C. (g) and (h) Mean state for CERA‐20C (dark blue) and SEAS5‐20C (red) for two distinct 30‐year hindcast periods as a function of season (CERA‐20C) or forecast target time (SEAS5‐20C). Data in (a)–(f) are estimated for 30‐year moving windows and plotted at the central year. The solid and dashed vertical lines in (a)–(d) indicate cross‐sectioned mean states shown in (g) and (h). Hatching in (e) and (f) indicates significant biases at *α* = 0.05.

In order to investigate how the background ENSO state evolved throughout the hindcast period, Figure 3 shows variations of moving 30‐year window mean NINO3.4 SST conditions in CERA‐20C (Figures 3a and 3b) and in the SEAS5‐20C hindcasts for November (Figure 3c) and May (Figure 3d) start dates. The vertical axis describes the seasonal cycle of CERA‐20C (note that Figures 1a and 1b ) show exactly the same data but plotted differently for convenience to compare with the model forecasts) and the lead time for the forecasts (Figures 3c and 3d). The observed absolute NINO3.4 SSTs experienced substantial changes throughout the twentieth century with temperatures in boreal winter being coldest in a period from the 1940s to 1970s and a warming during both summer and winter from the 1970s onward.

In contrast, SEAS5‐20C SSTs were warmest in the period mid‐1930s to mid‐1950s for a range of lead times with a cooling trend in the warm and cold season thereafter (Figures 3c and 3d). Displayed as biases of SEAS5‐20C with respect to CERA‐20C in Figures 3e and 3f, it becomes very apparent that the model has a significant cold bias for all lead time and both start dates during the most recent well‐observed decades, but suffers from a substantial and significant warm bias in boreal autumn and winter during the mid‐Century periods.

An illustration of the changes in the observed seasonal cycle is shown with the dark blue lines in Figures 3g and 3h which are vertical cross‐sections of Figures 3a and 3b for two distinct climate periods. The colder winter SSTs during 1930–1959 (dashed) compared to 1979–2008 (solid) are not reproduced in the hindcasts (red curves) which show much warmer SSTs in the winter season during the earlier period compared to the later period.

**Figure 4:**
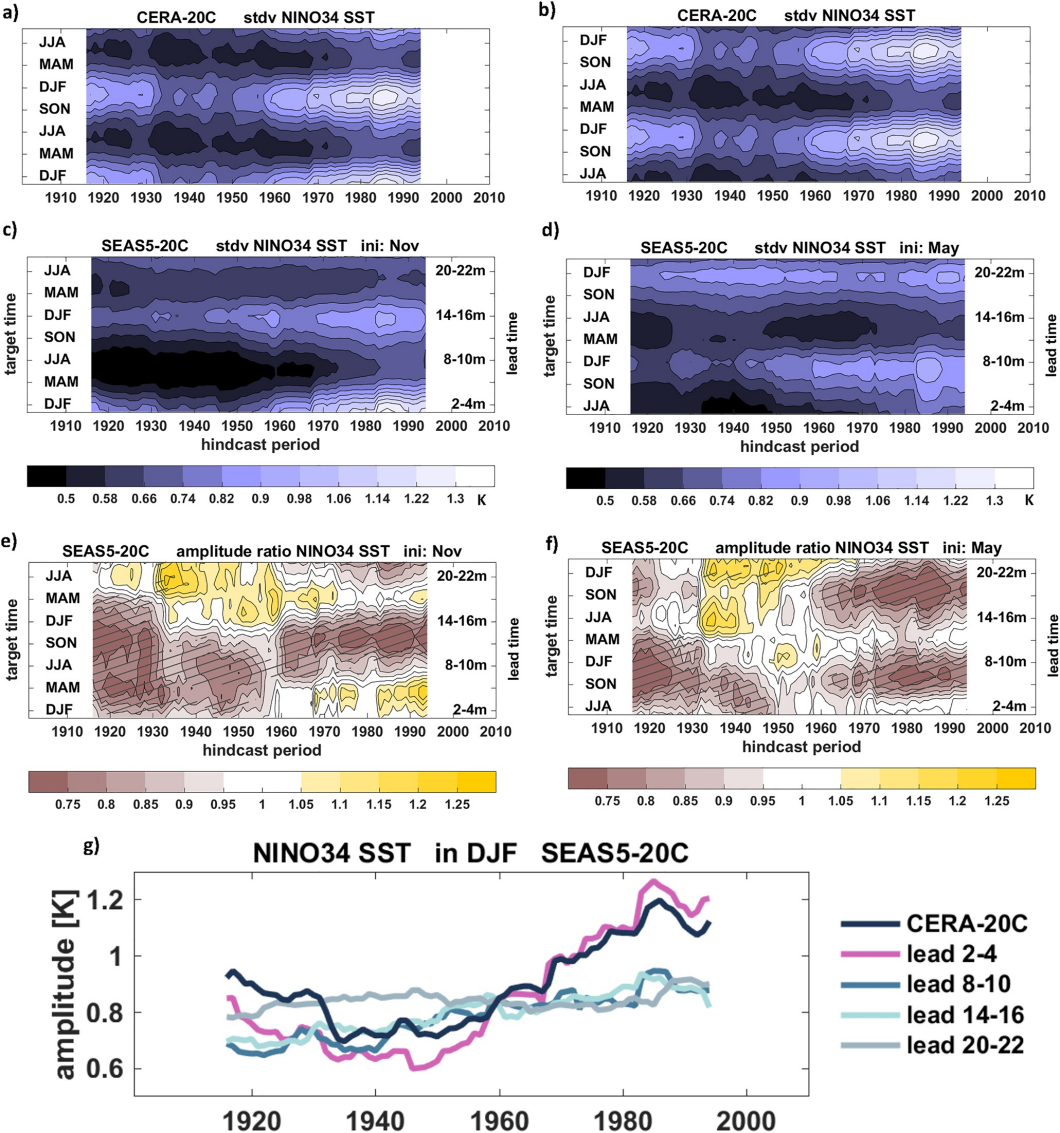
NINO3.4 SST standard deviation (amplitude) variations in CERA‐20C and SEAS5‐20C as a function of hindcast period and season/forecast lead time for 1 November (left) and 1 May (right) forecast start dates. (a) and (b) CERA‐20C. (c) and (d) SEAS5‐20C. (e) and (f) Amplitude ratio SEAS5‐20C to CERA‐20C. (g) Time series of amplitude in DJF for CERA‐20C (black) and the SEAS5‐20C forecasts with different lead times in months (colored lines). Data in (a)–(d) are estimated for 30‐year moving windows and plotted at the central year. Hatching in (e) and (f) indicates amplitude ratios significantly different from 1 at *α* = 0.05.

In addition to these variations of the SST mean state, the ENSO amplitude as a measure of interannual ENSO variability, or signal strength, also exhibits strong variations during the hindcast period (Figure 4). Here, the ENSO amplitude is defined as the standard deviation of the SSTs during 30‐year moving windows (for the model hindcasts the standard deviation is computed from all individual ensemble members). In the CERA‐20C reanalyses, the ENSO amplitude during the mature ENSO season DJF was at a minimum during the 1930s to 1960s, followed by a strong positive trend until the early 2000s (see also dark line in Figure 4g).

Encouragingly, SEAS5‐20C reproduced this overall behavior well for the short seasonal forecast lead times when initialized in November (Figure 4c and purple line in Figure 4g). It fails, however, to produce any noticeable long‐term variations of the ENSO signal for longer lead times (Figure 4d and lighter‐colored lines in Figure 4g) and thus substantially underestimates ENSO variability during the second half of the century. The amplitude ratio of SEAS5‐20C (defined as the ratio of the standard deviation in SEAS5‐20C to the standard deviation in CERA‐20C) is displayed in Figures 4e and 4f. The good agreement of the November initialized forecast amplitudes from the 1950s onwards before the spring barrier can clearly be seen (Figure 4e), whereas for longer lead times and the first half of the century the forecast amplitude is significantly smaller than in the reanalysis. In the May initializations, the amplitudes rapidly drop during the first month of the forecast before the 1950s, while in the second half of the hindcast period they are consistent with CERA‐20C for approx. three to four months but become significantly damped for the boreal autumn and winter seasons. For both start dates, however, ENSO forecast amplitudes are too strong at the longest lead times during the 1930s to 1950s.

## 5 Discussion and Conclusions

New multiannual reforecasts of the twentieth century to study the predictability of ENSO have been presented. The novelty of these experiments lies in the combination of the extensive length of the reforecast period (110 years) with the long forecast lead time of 2 years in a fully coupled and initialized state‐of‐the‐art global circulation model. Two reforecast start dates in May and November each year from 1901 to 2010 with an ensemble of 10 members are available.

A moving‐window analysis of correlation forecast skill shows that distinct periods of enhanced skill stand out against extended multi‐decadal epochs of reduced skill (Figure 1). ENSO prediction skill for DJF from initialization in November is steadily high from the 1950s onwards, in agreement with findings of Huang, Shin, et al. (2017) and Weisheimer et al. (2020). The skill temporarily drops during the periods centered between 1930–1950 and regains high levels in the first two decades of the century (see also Chen et al., 2004; Liu et al., 2021), a behavior that is also reflected on longer forecast lead times.

The reduced level of forecast skill seen before the well‐observed recent period raises the question whether fewer ocean observations in earlier periods might be the fundamental cause. The fact that skill is similarly high during the early decades of the twentieth century contradicts this suggestion. The interim skill reduction cannot simply be attributed to the inferior quality of the ICs because the skill reached equally high levels at the beginning of the twentieth century, when observations of the ocean were sparse. Rather, the low‐skill period broadly coincides with years when (a) the discrepancies in the SST mean state and seasonal cycle in observations and the model are largest with the model developing a strong warm bias and weakened seasonal cycle (Figure 3), (b) the observed ENSO amplitude is very weak (Figure 4), and (c) the persistence of observed SST anomalies is low (Figure 1h).

The observational coverage of the tropical Pacific saw a drop during the 1940s (Huang et al., 2020) which raises the question whether the increased uncertainty in the ICs impacted the forecast skill during this period. In order to estimate the sensitivity of our results to the specific SST product used, we repeated our analysis using the ERSSTv5 (Huang, Thorne, et al., 2017) data set for verification. A comparison of the ERSSTv5 and CERA‐20C (HadISST) data in terms of NINO3.4 SST mean state and variability and the corresponding model performance for biases and skill can be found in Figures S3–S5 in Supporting Information S1. The agreement in forecast skill suggests that these two observational data sets might share similarities in their methodology and in situ data. However, the lack of input data sets over the tropical Pacific precludes being able to definitively rule out related errors. On the other hand, the overall very good agreement of forecast skill and its multi‐decadal modulation implies that reduced ENSO skill during the 1930s to 1950s is not purely a result of the increased uncertainties in the observations or a potential temporal data issue in HadISST during the 1930s to 1950s. Rather, the robust findings using two different verification data sets and the atmospheric manifestation of ENSO through the SOI (see Figures 1c and 1d) strongly point toward a genuine attribute of predictive skill arising from changes in the mean state and variability.

The interpretation of the periods of decreased skill as a manifestation of multi‐decadal variations in predictability associated with modulations in the strength of the ENSO signal is fully consistent with the delayed oscillator mechanism discussed in Kirtman and Schopf (1998). Capturing these variations in background state and associated impacts on ENSO variability and (perfect model) predictability is still a major challenge for state‐of‐the‐art general circulation models used in seasonal and decadal predictions. Indeed, there are large discrepancies between the decadal modulations of SEAS5‐20C real and perfect model skill, which need to be further understood. A simple analysis with detrended time series (Figure S6 in Supporting Information S1) indicates that the increased perfect model skill during the latest period of the record cannot be attributed to the presence of trends in SSTs (L’Heureux et al., 2022).

By contrasting the varying characteristics of ENSO mean state and variability throughout the hindcast period as diagnosed in Figures 3 and 4 with the skill variations reported in Figure 1, we find that the overall higher levels of forecast skill during the second half of the twentieth century found in the November hindcasts concur with periods of strong ENSO amplitudes in both the observations and the shorter forecasts ranges, lending support for the suggested link between ENSO variability and predictability (Capotondi et al., 2015; Chen et al., 2004; Gu & Philander, 1997; Suarez & Schopf, 1988; Zhao et al., 2016) which reflects a reduction of the signal‐to‐noise ratio (Huang et al., 2021; Kumar, 2009).

The sharp decrease in prediction skill during boreal spring, known as the boreal spring predictability barrier (Liu et al., 2019; Webster & Yang, 1992), is a critical challenge for ENSO predictions on longer time ranges. We find that forecast skill beyond the spring barrier varies between decades of substantially longer skill from the 1980s onward (see also Balmaseda et al., 1995; Fang et al., 2019) and prolonged periods of abrupt and significant skill drops during spring. Once the forecast skill extends beyond the first boreal spring barrier, the predictability limit is much enhanced. For example, Dunstone et al. (2016) reported significant ENSO skill into the second winter and Mayer and Balmaseda (2021) found useful information of SEAS5 in year 2 in the specific case of the strong 1997/98 and 2014–2016 ENSO periods. Our results provide further support for the feasibility of skillful ENSO forecasts up to 18 months.

Questions as to changes in the spatial ENSO characteristics and which forced or unforced mechanisms exactly govern periods with enhanced long‐range skill (Timmermann et al., 2018; Wengel et al., 2018) will require more detailed research in the future. We conclude by noting that these extensive reforecast experiments can potentially also become useful data sets to train deep learning algorithms and other machine learning techniques (Ham et al., 2019; LeCun et al., 2015; Rasp et al., 2018) in order to facilitate the development of powerful and reliable dynamical long‐range forecasting systems for the future.

## Supporting information

Supporting Information S1

## Data Availability

CERA‐20C data is available through Laloyaux et al., 2018. The SEAS5‐20C reforecast experiment data used in this study (ECMWF, 2021) are available under a Creative Commons Attribution 4.0 International license (CC BY 4.0). To view a copy of this license, visit https://creativecommons.org/licenses/by/4.0/.
