# Supplementary material for: Variability of ENSO Forecast Skill in 2‐Year Global Reforecasts Over the 20th Century
Source: Geophys Res Lett. 2022 May 18;49(10):e2022GL097885. doi: 10.1029/2022GL097885 (PMC9285585; doi:10.1029/2022GL097885)
Supplement: Supplementary file 1 — Supporting Information S1 [file GRL-49-0-s001.docx]

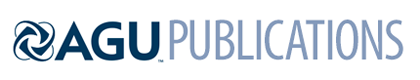


*Geophysical Research Letters*

Supporting Information for

**Variability of ENSO forecast skill in 2-year global reforecasts over the 20^th^ Century**

Antje Weisheimer^1,2^, Magdalena A. Balmaseda^1^, Tim N. Stockdale^1^, Michael Mayer^1,3^, S. Sharmila^4,5^, Harry Hendon^5,6^ and Oscar Alves^5^

^1^European Centre for Medium-Range Weather Forecasts (ECMWF), Reading, UK

^2^Univeristy of Oxford, Physics Department, National Centre for Atmospheric Science (NCAS), Oxford, UK

^3^Department of Meteorology and Geophysics, University of Vienna, Vienna, Austria

^4^Centre for Applied Climate Sciences, University of Southern Queensland, Toowoomba, Australia

^5^Bureau of Meteorology, Melbourne, Australia

^6^Monash University, Melbourne, Australia

**Contents of this file**

Figures S1 to S6

**Introduction**

This Supporting Information contains additional yet not crucial plots from the diagnostics carried out of the forecast data described in the main manuscript.

Figure S1. ENSO ensemble-mean correlation skill of SEAS5-20C with ERSSTv5 (left) and CERA-20C (right) as a function of hindcast period on the horizontal axis and forecast lead time on the vertical axis. a+b) 1st November initialization. c+d) 1st May initialization. Hatching indicates non-significant skill at 𝛼=0.05. Plots for CERA-20C on the right have been taken from the main manuscript and are repeated here for ease of comparison with the ERSSTv5 data on the left.

Figure S2. ENSO ensemble-mean correlation skill of SEAS5-20C as a function of hindcast period on the horizontal axis and forecast lead time on the vertical axis for 20-year (left) and 30-year (right) moving windows for 1st November initialization. a+b) NINO3.4 SST. c+d) Southern Oscillation Index (SOI). e+f) NINO3.4 SST perfect model skill. All plots for the 30-year windows on the right have been taken from the main manuscript and are repeated here for ease of comparison with the 20-year windows on the left. Hatching indicates non-significant skill at 𝛼=0.05.

Figure S3. Similar to Figure S2 but for 1st May forecast start dates.

Figure S4. NINO3.4 SST characteristics in ERSSTv5 (left) and CERA-20C (right) as a function of historical/hindcast period and season/forecast lead time for 1st November forecast start dates. a+b): Mean-state variations in the SST observational data sets. c+d): Bias of SEAS5-20C. e+f) Standard deviation (amplitude) variations in the SST observational data sets. g+h) Amplitude ratio of SEAS5-20C. Hatching in c+d) indicates significant biases at 𝛼=0.05. Hatching in g+h) indicates amplitude ratios significantly different from 1 at 𝛼=0.05. All plots for CERA-20C on the right have been taken from the main manuscript and are repeated here for ease of comparison with the ERSSTv5 data on the left. Colorbars are from the main manuscript optimized for CERA-20C data.

Figure S5. Similar to Figure S4 but for 1st May forecast start dates.

Figure S6. NINO3.4 perfect model skill in SEAS5-20C based on non-detrended timeseries (a) and detrended timeseries (b) for 1st November forecast start dates. In b) the linear trend from 1901 to 2010 in the reanalysis and forecast data has been removed.
